# 

**Published:** 1815-05

**Authors:** 


### MONTHLY CATALOGUE OF MEDICAL BOOKS.

ON Gun-shot Wounds of the Extremities requiring the Different Operations of Amputation, with their after-treatment; establishing the Advantages of Amputation on the Field of Battle to the Delay usually recommended, &c. &c. &c. with Four Explanatory Plates. By G. J. Guthrie. 8vo.—Longman and Co.

Practical Explanation of Cancer in the Female Breast. By John Rodman, M.D. 8vo.—Underwood.

Part of the Introductory Lecture for the Year 1815, exhibiting some of Mr. Hunter's Opinions respecting Diseases. By John Abernethy, F.R.S. 8vo. Longman and Co.

Observations and Reflections on the Bill now in progress through the House of Commons, for better Regulating the Medical Profession as far as regards Apothecaries. By R. M. Kerrison. 8vo. —Longman and Co.

Fasciculus II. of a Series of Engravings of Cutaneous Diseases, illustrative of the principal Genera and Species described in the Practical Synopsis. By Dr. Bateman. 4to.—Longman and Co.

A Sketch of the New Anatomy and Physiology of the Brain and Nervous System of Drs. Gall and Spurzheim, considered as comprehending a complete System of Zoonomy; with Observations on its Tendency to the Improvement of Education, of Punishment, and of the Treatment of Insanity. Reprinted from the Pamphleteer, with additions. By T. Forster, F.L.S. 5s.

---

### *NOTICES TO CORRESPONDENTS.*

"A CONSTANT READER" *is referred to the Printer,* (23, Bartholomew-Close,) *for the Explanation he requires.*

*The Revie[illegible] Dr. Spurzheim has been delayed on account of some inaccuracies in the Diagram, which the Engraver has not yet corrected.*

*Dr. Adams's Paper on Goitre and Cretin arrived too late for insertion this Month.*

*We have also been favoured with Communications from Dr. Haxby, Dr. Robertson, Mr. Atkinson, Mr. Smerdon, &c. &c.*

---

☞ *Communications (post paid) are thankfully received, and should be addressed to the Editors, No. 1, Paternoster Row; and, if intended for Insertion in the subsequent Number, they ought to come to hand before the 12th of the Month.*

